# Characterization of patients with acutely decompensated cirrhosis who received care in different highly complex emergency services of Medellín, Colombia.

**DOI:** 10.7705/biomedica.6963

**Published:** 2023-12-29

**Authors:** Juan Luis Vélez, Andrea Pérez, Juan David Blanco, Marie Claire Berrouet, Lorena Valencia, Sofía Soto, Ana Sofía Ramírez, Víctor Martínez, Juan Luis Gallego, Julia Jaillier

**Affiliations:** 1 Facultad de Medicina, Universidad CES, Medellín, Colombia Universidad CES Universidad CES Medellín Colombia; 2 Servicio de Toxicología Clínica, Hospital General de Medellín “Luz Castro de Gutiérrez”, Medellín, Colombia Hospital General de Medellín Luz Castro de Gutiérrez Hospital General de Medellín “Luz Castro de Gutiérrez” Medellín Colombia; 3 Fundación Universitaria San Martín, Facultad de Medicina, Sabaneta, Colombia Fundación Universitaria San Martín Fundación Universitaria San Martín Sabaneta Colombia

**Keywords:** liver cirrhosis, emergencies, emergency service, hospital, health resources, public health surveillance, health expenditures, cirrosis hepática, urgencias médicas, servicio de urgencias en hospital, recursos en salud, vigilancia en salud pública, gastos en salud

## Abstract

**Introduction.:**

Cirrhosis is one of the ten leading causes of death in the Western hemisphere and entails a significant cost of health care.

**Objective.:**

To describe the sociodemographic, clinical, and laboratory characteristics of patients older than 18 years who received care for acute decompensation of cirrhosis in the emergency services of three highly complex centers in Medellín, Colombia.

**Materials and methods.:**

This was an observational retrospective cohort study from clinical records. The results were analyzed by frequency measures and represented in tables and graphics.

**Results.:**

In total, 576 clinical records met the inclusion criteria; 287 were included for analysis, and 58.9% were men, with an average age of 64 (± 13.5) years. The most frequent causes of cirrhosis were alcohol intake (47.7%), cryptogenic or unspecified etiology (29.6%), and non-alcoholic fatty liver disease (9.1%). The main reasons for visiting the emergency department were the presence of edema and/or ascites (34.1%), suspicion of gastrointestinal bleeding (26.5%), abdominal pain (14.3%) and altered mental status (13.9%).

The most frequent clinical manifestations of an acute decompensation of cirrhosis were ascites (45.6%), variceal hemorrhage (25.4%), hepatic encephalopathy (23.0%), and spontaneous bacterial peritonitis (5.2%).

During their treatment, 56.1% of the patients received intravenous antibiotics; 24.0%, human albumin; 24.0%, vasoactive support, and 27.5%, blood products; 21.3% required management in an intensive or intermediate care unit, registering 53 deceased patients for a mortality of 18.5%.

**Conclusion.:**

Patients who consult the emergency services due to acute decompensation of cirrhosis demand a high amount of health resources, frequently present associated complications, and a high percentage requires management in critical care units and shows a high in-hospital mortality rate.

## Resumen

**Introducción.:**

La cirrosis hace parte de las diez primeras causas de muerte en el hemisferio occidental y acarrea un importante costo en salud.

**Objetivo.:**

Describir las características sociodemográficas, clínicas y de laboratorio, de los pacientes mayores de 18 años que recibieron atención por descompensación aguda de la cirrosis en los servicios de urgencias de tres centros de alta complejidad en Medellín, Colombia.

**Materiales y métodos.:**

Se trata de un estudio observacional de cohorte. Los resultados se analizaron mediante medidas de frecuencia, y se representaron en tablas y gráficas.

**Resultados.:**

En total, en 576 registros clínicos se cumplieron los criterios de inclusión; se incluyeron 287 para el análisis. El 58,9 % fueron hombres, con edad promedio de 64 (± 13,5) años. Las causas más frecuentes de cirrosis fueron: ingestión de alcohol (47,7 %), criptogénica o inespecífica (29,6 %) y enfermedad por hígado graso no alcohólico (9,1 %). Los principales motivos de consulta fueron: presencia de edemas, ascitis o ambas (34,1 %), sospecha de hemorragia digestiva (26,5 %), dolor abdominal (14,3 %) y alteración del estado mental (13,9 %). Los diagnósticos de complicación aguda más frecuentes fueron ascitis (45,6 %), hemorragia digestiva por várices esofágicas (25,4 %), encefalopatía hepática (23,0 %) y peritonitis bacteriana espontánea (5,2 %). El 56,1 % de los pacientes recibió antibióticos; el 24,0 %, albúmina humana; el 24,0 % medicamentos, y el 27,5 % hemoderivados. En el 21,3 % de los casos, se requirió hospitalización en la unidad de cuidados intensivos o en la de cuidados intermedios. Se registraron 53 decesos, para una mortalidad del 18,5 %.

**Conclusiones.:**

Los pacientes que consultan a los servicios de urgencias por una descompensación aguda de la cirrosis demandan una gran cantidad de recursos, frecuentemente presentan complicaciones asociadas, requieren manejo en unidades de cuidado crítico y evidencian una alta tasa de mortalidad.

Cirrhosis is a chronic liver disease that occurs as the final stage of progressive fibrosis that affects the hepatocyte and can be caused by multiple etiologies ^1^. Its current importance lies in being one of the top ten causes of death in the Western hemisphere ^2^, representing a significant decrease in life quality and expectancy, as well as a substantial increase in healthcare costs.

This public health problem is attributed to approximately 1’000,000 deaths per year ^3^^,^^4^, with a mortality rate that ranges from 48 to 68 cases per 100,000 people in European countries, and approximately 117.41 cases per 100,000 people in México ^5^^,^^6^. Among people with cirrhosis, the relative risk of death increases 4.7 times, and in cases of decompensation, up to 9.7 times ^7^. When acute decompensation of cirrhosis occurs, the 5-year survival decreases by 50% ^8^, with a 30% mortality rate at one month and 70% at one year ^2^^,^^9^. Cirrhosis is a significant burden on the healthcare system, with the cost of end-stage care estimated to be between USD$ 12 and 23 billion annually in the United States and the annual cost of emergency services ranging from USD$ 375.65 to USD$ 4,145.09 per patient in Latin America ^10^.

Observational studies conducted in Colombia between 2014 and 2020 have shown that the most common acute complications of cirrhosis are variceal bleeding, ascites, and encephalopathy. The most common causes of cirrhosis were non-alcoholic fatty liver disease, alcohol use, and hepatitis C ^2^^,^^4^^,^^11^^,^^12^. The overall prevalence of decompensation is estimated at 60%, and the natural history of cirrhotic disease indicates that 42% of patients progress from a compensated to a decompensated state within 10 years ^13^.

Despite efforts to characterize this special population, there is still a gap in knowledge, particularly regarding the acute presentation of decompensation and the sociodemographic, clinical, and laboratory characteristics associated with the main reasons for emergency department visits for these patients. Understanding this information facilitates decision-making in the triage process, hospital disposition, resource allocation, outcome prognosis, and risk prevention. From a public health perspective, it allows for the development of care and promotion and prevention strategies aimed at responding to the specific needs of this patient group.

The present study aimed to describe the sociodemographic, clinical, and laboratory characteristics of patients over 18 years of age who received care for acute decompensation of cirrhosis in the emergency departments of three highly complex centers in Medellín, during the period 2015 to 2019.

## Materials and methods

This article follows the recommendations for reporting observational epidemiological studies from the STROBE statement ^14^. A retrospective, longitudinal, and descriptive cohort study was conducted in three high- complexity hospital centers in Medellín, one of which is a governmental hospital (*Hospital General “Luz Castro de Gutiérrez”*) and the other two are privately managed (*Clínica CES* and *Clínica Las Américas* - AUNA).

Medellín is in a metropolitan area with an approximate population of 4,000,000 inhabitants ^15^ and is the reference center for approximately 12% of the Colombian population ^16^, estimated at 48’258,494 people ^17^.

The study included all records of care provided between January 1^st^., 2015, and December 31^st^., 2019, to patients:

1. over 18 years of age,
2. with an established diagnosis of liver cirrhosis, and
3. who consulted the emergency department of the participating centers due to acute decompensation of cirrhosis, identified from the discharge ICD-10 diagnosis codes related to cirrhosis and its etiologies (K746, K703, K745, K743, K744, K717).

Patients

1. who expressed disagreement with the use of their personal data,
2. in whom the diagnosis of cirrhosis was ruled out based on their medical history,
3. in whom it was not possible to identify an acute complication of cirrhosis as the reason for the emergency department visit, and
4. those with medical records with 20% or more of incomplete predefined variable information were excluded. After applying the above criteria, 576 eligible clinical records were obtained, of which 287 were included in the analysis.

Sociodemographic, clinical, and laboratory variables were defined. Sociodemographic variables included gender and age, which were grouped by two decades starting from 30 years. Clinical characteristics were grouped by chief complaint, etiology of cirrhosis, Child-Pugh score, MELD score, and type of acute decompensation. In addition, the following outcomes were measured dichotomously: use of vasoactive drugs, blood transfusions, antibiotic therapy, and/or albumin; need for admission to the intensive care or intermediate care unit and death during hospitalization.

Relevant laboratory variables for patients with cirrhosis were collected: hemoglobin, leukocytes, and platelets, hepatic and renal biochemistry; all of them described in internationally accepted units. The recorded values corresponded to the laboratory tests performed at the time of admission to the emergency department with a margin of up to the following 72 hours. For the variable of albumin level, the margin was allowed to include the last registered value in the previous 21 days before admission in case it was not available in the first 72 hours from the emergency department visit.

The measured variables were manually extracted from the data obtained from the electronic medical records. The obtained data were collected in an organized manner in an instrument developed in Microsoft Excel™. Dropdown lists with response options were created for qualitative variables, and cell formats were adapted to predetermined value ranges for quantitative variables. Ten percent of the collected data was double-checked by different investigators. A pilot test and standardization of the instruments were performed before starting data collection. All patients who met the inclusion and exclusion criteria were included in the study; therefore, no sample calculation was performed.

The data collected in the instruments were cleaned and unified in Microsoft Excel, then exported and analyzed using Jamovi statistical software, version 1.2. Absolute and relative frequency measures were calculated for qualitative variables. Measures of central tendency were calculated for quantitative variables, and the behavior of mortality was analyzed by calculating the rate. Total results were organized through frequency tables. The most relevant results were represented by bar and pie charts.

The study protocol was submitted to the Human Research Ethics Committee at the Universidad CES for approval. The protocol was approved and recorded in Act 167 under project code: Ae-717. In addition, the study protocol was also approved by the respective ethics committees of each participating institution. The study was classified as a low-risk study based on Resolution 8430 of 1993 issued by the *Ministerio de Salud de Colombia*.

## Results

**Figure 1:**
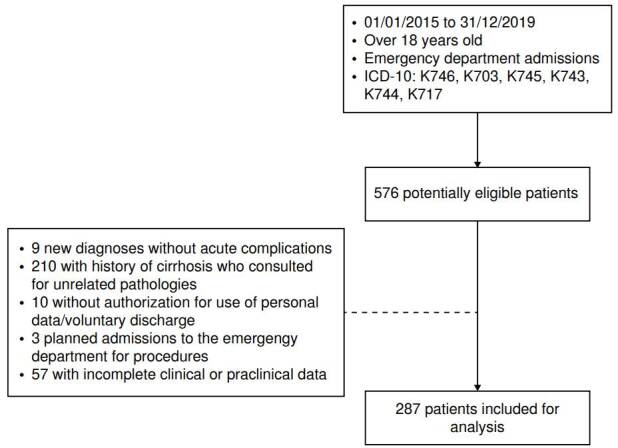
Inclusion criteria and reasons for exclusion of participants

From the selected population, 576 clinical records were obtained that met the inclusion criteria, of which 287 were included for analysis. The reasons for exclusion of the remaining records included scheduled admission for emergency procedures (1%), uncomplicated de novo diagnosis (3.1%), voluntary discharge or non-authorization of the use of personal data (3.4%), incomplete data in the medical history (19.7%), and a diagnosis other than an acute complication of cirrhosis as the cause of consultation to the emergency department (72%) (figure 1).

About the patients, the mean age was 64 (± 13.5) years, 58.9% were male and 86% were over 50 years old. Regarding the etiologies of cirrhosis, alcohol consumption was the most described cause, accounting for 47.7% of cases. Other frequent etiologies were cryptogenic or unspecified etiology in 29.6% and fatty liver disease in 9.1%. Viral hepatitis accounted for 7.3% of cirrhosis cases, while biliary (primary or secondary) and autoimmune origin cirrhosis had a frequency of 3.1% each. There were no cases related to metal deposit disease or congenital enzyme deficiencies. Regarding the progression of liver disease, most patients presented with an advanced Child-Pugh score, with 38.7% of patients in score B and 47.7% in score C, with an average MELD score of 18 (± 8) points and a median of 17 points.

**Figure 2:**
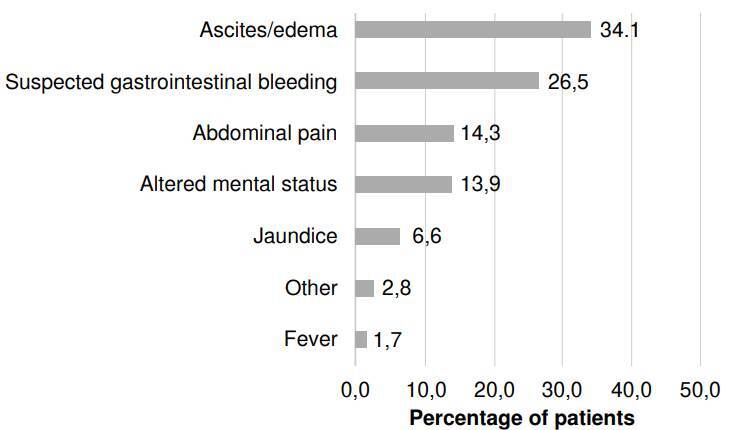
Reasons for visiting the emergency department in patients with an acute decompensation of cirrhosis

The main reasons for emergency department visits (figure 2) or chief complaints found in the study were the presence of edema and/or ascites (34.1%), suspicion of gastrointestinal bleeding (26.5%), abdominal pain (14.3%), altered mental status (13.9%), jaundice (6.6%), other unspecified reasons (2.8%), and fever (1.7%).

**Figure 3:**
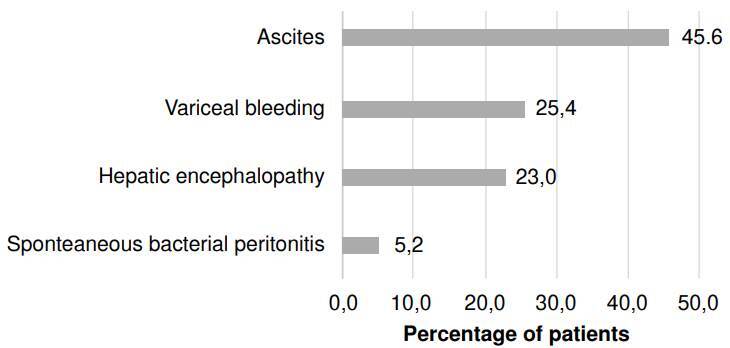
Acute complication of cirrhosis as the primary or main diagnosis during index hospitalization

**Figure 4:**
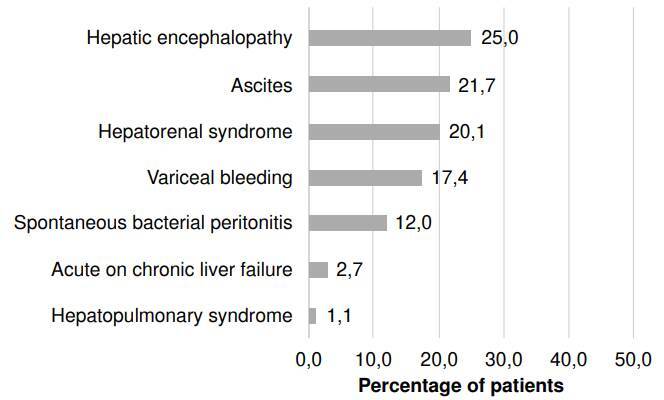
Diagnoses of acute complications of cirrhosis associated with the primary or main diagnosis

The main complication was determined to be the diagnosis directly related to the reason for consultation. The most frequent main diagnoses of acute complications of cirrhosis corresponded to the presence of ascites (45.6%) and variceal hemorrhage (25.4%), followed by hepatic encephalopathy and spontaneous bacterial peritonitis, with a frequency of 23.0% and 5.2%, respectively (figure 3). In 43.6% of cases, more than one acute complication of cirrhosis was identified concomitantly during hospitalization (figure 4). The most frequently associated acute complications with the main diagnosis were hepatic encephalopathy (25.0%), ascites (21.7%), hepatorenal syndrome (20.1%), variceal hemorrhage (17.4%), spontaneous bacterial peritonitis (12%), acute-on-chronic liver failure (2.7%), and hepatopulmonary syndrome (1.1%) (table 1) (figure 4).

**Table 1:** Qualitative variables: Sociodemographic and clinical characteristics

		n=287	%
Gender			41.1
	Female	118	58.9
	Male	169	
Age groups (years)			1.4
	18 to 30	4	12.5
	31 to 50	36	50.5
	51 to 70	145	34.5
	71 to 90	99	1.0
	Over 90	3	
Child-Pugh score			4.9
	NC	14	8.7
	A	25	38.7
	B	111	47.7
	C	137	
Reasons for visiting the emergency department or chief complaints			
	Fever	5	1.7
	Other	8	2.8
	Jaundice	19	6.6
	Altered mental status	40	13.9
	Abdominal pain	41	14.3
	Suspected gastrointestinal bleeding	76	26.5
	Ascites/edema	98	34.1
Etiology of cirrhosis			
	Secondary biliary cirrhosis	2	0.7
	Hepatitis C	4	1.4
	Primary biliary cirrhosis	7	2.4
	Autoimmune hepatitis	9	3.1
	Hepatitis B	17	5.9
	Nonalcoholic hepatic steatosis	26	9.1
	Cryptogenic or no specified	85	29.6
	Alcohol consumption	137	47.7
Primary or main diagnosis			
	Acute on chronic liver failure	2	0.7
	Spontaneous bacterial peritonitis	15	5.2
	Hepatic encephalopathy	66	23.0
	Variceal bleeding	73	25.4
	Ascites	131	45.6
Associated diagnosis			
	Hepatopulmonary syndrome	2	1.1
	Acute on chronic liver failure	5	2.7
	Spontaneous bacterial peritonitis	22	12.0
	Variceal bleeding	32	17.4
	Hepatorenal syndrome	37	20.1
	Ascites	40	21.7
	Hepatic encephalopathy	46	25.0
	No associated diagnosis	162	56.4
Admission to critical care unit			
	Critical care admission	61	21.3
	General wards	223	77.7
Condition at hospital discharge			
	Deceased	53	18.5
	Alive	234	81.5
Use of hospital resources			
	Vasoactive drugs	69	24.0
	Albumin	69	24.0
	Blood derived products	79	27.5
	Antibiotics	161	56.1

**Figure 5:**
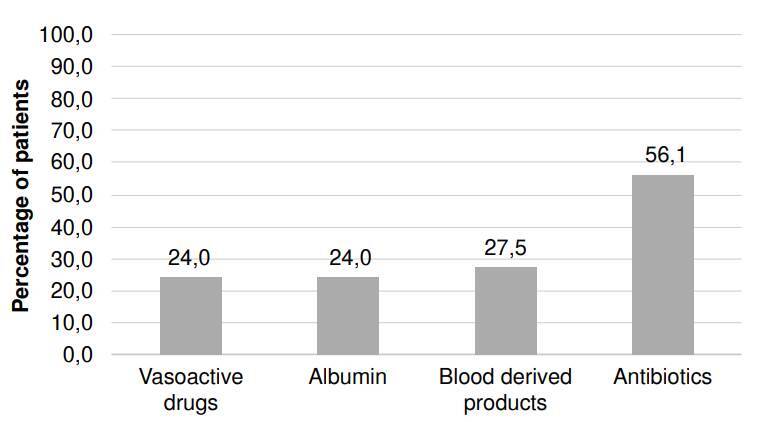
Type of health resource and percentage of its use in patients with acute decompensation of cirrhosis who visited the emergency department

Regarding the use of hospital resources, 56.1% of patients received intravenous antibiotics, 24.0% received human albumin, 24.0% received vasopressor support, and 27.5% required the use of blood products (figure 5). The average length of hospital stay was 9 (± 8) days, with a median of 7 days.

In general, laboratory findings demonstrated that patients who were admitted through emergency services had average values below the expected normal range for hemoglobin, platelet count, and albumin, and above normal values for transaminase, INR, and total bilirubin measurements. Among all clinical records included absence of paraclinical data was found in varying percentages of the total patients from initial paraclinical assessment in the emergency service. For the transaminases value, approximately 17% of patients did not have the data, 16% for serum albumin, 14% for blood urea nitrogen levels, and 11% for total bilirubin values (table 2).

**Table 2:** Quantitative variables: Laboratory values, End-Stage Liver Disease (MELD) model score, and days of hospital stay are included.

	HB	LEU	PLT	CR	BUN	ALT	AST	BT	ALB	INR	MELD	DE
n= 287	286	284	284	281	246	238	238	255	241	274	239	287
Prom,	11,0	8.254	137.334	1,16	26,8	78,1	116	5,09	2,68	1,7	18	9
P50	11,4	6.550	119.000	0,85	20,6	39,5	64	2,43	2,6	1,49	17	7
Min	3,4	680	1.240	0,3	3,5	7,5	13,1	0,4	1,45	0,9	6	6
Max	18,6	66.100	598.000	6,87	135	2750	3074	35,4	4,45	8,03	48	79
SD	2,8	6.325	90.471	1,0	20,1	226	241	6,5	0,5	0,8	9	8

HB: hemoglobin in g/dl; LEU: leukocyte count per mm^3^; PLT: platelet count per mm^3^; RR: creatinine in mg/dl; BUN: blood urea nitrogen in mg/dl; ALT: alanine aminotransferase in U/L; AST: aspartate aminotransferase in U/L; BT total bilirubin in mg/dl; ALB: plasma albumin in g/dl; INR: ratio of internationally normalized prothrombin time; MELD: Model for End-stage Liver Disease; DE: days of hospital stay; Prom: average; P50: 50th percentile or median; Min: minimum value; Max: maximum value; SD: standard deviation

Of the 287 patients included in the analysis, 21.3% required management in intensive or intermediate care units, with 53 registered deaths, corresponding to an in-hospital mortality rate of 18.5% of analyzed admissions. In a follow-up time of 2,570 days/patient, this translates to a mortality rate of 2 deaths per 100 days/patient.

## Discussion

In Colombia, the epidemiological behavior of cirrhotic patients has been described through retrospective and prospective studies aimed at characterizing the population receiving care in hepatology and gastroenterology outpatient services, as well as hospitalization in general wards ^2^^,^^4^^,^^11^^,^^12^^,^^18^. The relevance of this study lies in the fact that, after an extensive literature review and to our knowledge, it is the first and largest of its kind, both locally and in Latin America, to describe the presentation of acutely decompensated patients in the emergency department based on the reasons for consultation or chief complaints.

A study conducted in Bogotá by Prieto *et al*. ^11^ which focused on the relationship between the etiology of cirrhosis and the presence of acute decompensations that led to seeking emergency care, described nonalcoholic steatohepatitis as the main etiology of cirrhosis, with ascites, variceal bleeding, and hepatocellular carcinoma being the main diagnoses of acute decompensation of cirrhosis. This contrasts partially with our findings, since while both ascites and variceal bleeding coincide as the two main complications, the predominant etiology in this study is alcohol consumption, five times greater than non-alcoholic steatohepatitis.

In 2020, a retrospective study from a single specialized center in Medellín described the characteristics of a cohort of 78 cirrhosis patients hospitalized due to a diagnosis of spontaneous bacteremia ^2^. This study found that the main etiology of cirrhosis associated with spontaneous bacteremia was cryptogenic, followed by non-alcoholic steatohepatitis. It also reported a 30- day mortality of 11.5%. In our study, the diagnosis of spontaneous bacteremia as an acute complication of cirrhosis was not identified in the clinical records, probably due to underreporting and the inclusion of this diagnosis within other infectious complications. However, non-alcoholic steatohepatitis and cryptogenic cirrhosis were found among the three main etiologies.

Regarding the etiology of cirrhosis, a study conducted in the Caribbean region of Colombia found that hepatitis C infection was the main cause of the disease, followed by non-alcoholic fatty liver disease ^4^, which contrasts with the results of our study where alcohol consumption was identified as the etiology in nearly half of all cases of cirrhosis and viral hepatitis in only 7.3%, highlighting the regional epidemiological differences within the same country. Although in our study cryptogenic etiology surpassed non-alcoholic fatty liver disease as the cause of cirrhosis, it is possible that this is due to underreporting and the absence of a complete study of various cardiovascular risk factors in patients.

Likely, the findings of this study have a closer relationship with the study conducted by Giraldo-Montoya *et al*. in the Risaralda department and the central region of the country ^12^, where the epidemiological behavior of patients with cirrhosis was quite similar to that found in the present study, documenting that the main underlying etiology of cirrhosis was alcohol, with ascites being the most frequently described complication, followed by variceal upper gastrointestinal bleeding and encephalopathy. Additionally, in that study, the presence of encephalopathy or ascites had an association with death with statistical significance.

It is important to interpret the results on the etiology of cirrhosis in this study in light of the findings of the *Encuesta Nacional de Consumo de Sustancias Psicoactivas 2019,* Colombia (ENCSPA) ^19^, which described alcohol consumption as a public health problem in Colombia: 84% of people between the ages of 12 and 65 have consumed alcohol at some point in their lives, 54.5% have done so in the last 12 months, and 30.1% in the last month. In addition, when describing alcohol consumption and prevalence by gender, men start consuming alcohol about 2 years earlier than women, a higher percentage start consuming it in the last year (25.9% vs. 19.0%), and they have a higher annual prevalence of consumption, at 63.1% for men and 46.6% for women. Specifically, for Medellin and the department of Antioquia, according to the ENCSPA, the annual prevalence of alcohol consumption is 53.3% and 53.7%, respectively. The foregoing results may account for the higher percentage of men than women, as well as the higher prevalence of alcohol-related cirrhosis, as evidenced in our study.

In our study, the main acute complications included ascites, variceal bleeding, and encephalopathy, with an in-hospital mortality rate of 18.5%, slightly lower than that reported in other national and Latin American studies, where it exceeds 20% ^20^. This could be explained because the outcome of “condition at hospital discharge”, from which the data on in-hospital mortality was obtained, was collected based on the condition at the time of discharge from the index hospitalization, without documenting the final outcome in case the patient was referred to a higher complexity center or an external critical care unit, including patients who may have died in another institution as a result of the index hospitalization within the percentage of surviving patients.

On the other hand, although the present study described the “presence of ascites or increased edema,” “suspicion of gastrointestinal bleeding,” “abdominal pain,” and “alteration of mental status” as the main reasons for emergency department visits among patients with decompensated cirrhosis, and for the experienced clinician, it would be easy to relate these complaints to a specific acute complication of cirrhosis, one in five patients included in the study presented more than one acute complication simultaneously.

It is noteworthy that hepatorenal syndrome was one of the three main complications associated with the primary diagnosis in 20.1% of cases, which is consistent with recent reports describing acute kidney injury occurring in up to 50% of hospitalized patients with cirrhosis ^21^^-^^23^. However, it should be acknowledged that due to the retrospective nature of this study and its reliance on clinical records, there are limitations in distinguishing whether the documented diagnosis of hepatorenal syndrome truly adhered to the diagnostic criteria ^21^ or if it was an acute kidney injury established by other causes. Based on these results, it would be advisable to actively search for additional complications in patients with decompensated cirrhosis who are admitted to emergency services.

Furthermore, this study demonstrates that patients presenting with acute decompensation of cirrhosis in emergency services require a significant amount of resources, including human, physical, and technical resources. More than half of the patients who consulted emergency services for acute decompensation of cirrhosis required the use of antibiotics, approximately one in three received blood products, and one in four required vasopressor support and human albumin infusion. The average hospital stay for these patients was 9 (± 8) days, and nearly 20% required admission to high- complexity units. Although it is considered that patients with cirrhosis who present with acute decompensation require multiple laboratory tests to calculate the current staging of the disease and thus identify disease progression and promptly refer the patient to specialized liver transplant centers ^8^^,^^24^^-^^26^, in our study, up to 15% of patients did not have the necessary laboratory tests to calculate this staging. This finding should be taken into consideration as an opportunity for improvement in patient care.

The findings of this study highlight the high degree of complexity of care required by patients with liver cirrhosis and raise the possibility of generating more studies that provide evidence on the health costs and quality of life of these patients, particularly when considering that up to 30.1% of patients with cirrhosis consult emergency services more than four times a year ^27^.

Our study presents several strengths. Among them, we highlight a significant number of clinical records included in the analysis when compared to previous studies with similar objectives. Additionally, the study included a population from three highly complex hospital centers, not specialized in the care of patients with liver disease, but attending patients with heterogeneous demographic characteristics, including one of the largest national public hospitals, which is also a regional reference institution for both the center and northwest regions of the country. This allows the obtained results to be more easily generalizable and thus contribute to knowledge about the natural history of acute decompensations of cirrhosis. Furthermore, the rigorous and individualized follow-up of clinical records by trained medical personnel, combined with standardized data collection formats, ensures their quality. However, several limitations are also evident, such as those inherent to the retrospective nature of the study, considering the exclusion of a significant percentage of patients from the analysis due to incomplete data in the clinical records.

In conclusion, the history of cirrhosis is a red flag for various chief complaints that prompt emergency department visits. Our cohort demonstrates that patients who seek emergency services for acute decompensation require a high amount of healthcare resources, frequently present with associated complications, require management in critical care units in a high percentage, and show a high rate of in-hospital mortality. The authors hope that the results of this study serve as a basis to highlight the importance of standardizing care processes to improve the quality of health services for this group of patients and to encourage measures aimed at preventing this disease in vulnerable populations.
